# Functional movement variability to maintain delivery speed in cricket fast bowling

**DOI:** 10.1002/ejsc.12045

**Published:** 2024-03-05

**Authors:** Max H. Andrews, Adam D. Gorman, Rian H. Crowther

**Affiliations:** ^1^ School of Human Movement and Nutrition Sciences The University of Queensland Brisbane Queensland Australia; ^2^ Sport Science Sport Medicine Unit Cricket Australia National Cricket Centre Brisbane Queensland Australia; ^3^ School of Exercise and Nutrition Sciences Queensland University of Technology Brisbane Queensland Australia; ^4^ QUT Sport Queensland University of Technology Brisbane Queensland Australia

**Keywords:** adaptability, biomechanics, cricket fast bowling, functional movement variability, skill acquisition

## Abstract

Fast bowling in cricket involves a complex multiarticular action performed at high speed. Functional movement variability plays an important role in helping the performer to maintain the consistency of performance outcomes, although this has not previously been examined in cricket fast bowling in relation to maintaining consistent delivery speed. Therefore, this study investigated how variability of delivery stride movement timings contributes to maintaining consistent bowling speed. Footage of six deliveries from 92 male fast bowlers were analyzed. Delivery speed was recorded, and the time of back foot contact, front foot contact and ball release were identified. The variability of movement timings and the delivery speed were determined using the coefficient of variation. Results showed greater variability in delivery stride movement timings compared to delivery speed. These findings suggest that bowlers may use movement variability to adjust their functional movement coordination pattern during the bowling action to achieve consistent bowling speed.

## Highlights

- Variability of movement timings may play an important functional role in assisting cricket fast bowlers to adapt to the constraints of the task to achieve a consistent performance outcome.
- Findings support previous research in skill acquisition by highlighting that variability in movement patterns is necessary for consistent performance outcomes.
- This study presents preliminary evidence that functional movement variability in cricket fast bowlers contributes to achieving consistent delivery speed, challenging traditional views on minimizing movement variability for successful performance outcomes.

## 1 INTRODUCTION

Movement variability and performance outcome consistency are crucial for skilled performance (Button et al., 2003; Fleisig et al., 2009). Traditional views of skill acquisition and motor control (Adams, 1971; Schmidt, 1975) link consistent performance outcomes to a consistent (invariant) technique or movement pattern (Davids et al., 2008; Handford et al., 1997; Newell & Corcos, 1993). Movement variability was initially seen as an error in the system, leading to inconsistent performance outcomes (Handford et al., 1997). However, there has been a recent shift toward viewing movement variability as functional (Bartlett et al., 2007; Davids et al., 2003). Thus, variable movement patterns leading to consistent performance outcomes demonstrate the motor system's flexibility and adaptiveness (Davids et al., 2015). Button et al. (2003) defined movement variability as “the degree of change in coordination patterns between trials” (p. 258). With the growing acceptance that trial‐to‐trial movement variations are necessary for success (Davids et al., 2015), further research is required to investigate the role of variability in movement coordination and its effect on performance (Langdown et al., 2012).

Greater movement pattern variability is associated with higher expertize (Davids et al., 2008; Sayers et al., 2017; Wilson et al., 2008). However, patterns of variability, not the magnitude, appear closely related to performance consistency (Button et al., 2003; Dias et al., 2014). In addition, movement variability progressively decreases throughout many tasks (Bootsma & van Wieringen, 1990; Horan et al., 2011; Morrison et al., 2014). For example, in table tennis forehand drives, racket movement variability decreases from the start of the drive to bat–ball contact (Bootsma & van Wieringen, 1990). Thus, the variability decreases as the drive nears bat–ball contact ensuring consistent contact (Bootsma & van Wieringen, 1990). The movement pattern likely varied in response to changes associated with the approaching ball, even though timing and direction were consistent at contact (Bootsma & van Wieringen, 1990). Golf swings also show reduced variability toward the end, suggesting that performers coordinate the movement of their joints throughout the swing to home‐in on a specific endpoint (Morrison et al., 2014; Sayers et al., 2017). Baseball pitchers also appear to reduce variability throughout their throwing action (Matsuo et al., 2001), potentially in response to early inconsistencies in movement timings, to produce a consistent outcome (Matsuo et al., 2020). Therefore, high variability at the start of movements may enable precise adjustments to external and internal perturbations (Bootsma & Peper, 1992).

Cricket fast bowlers consistently deliver the ball at high speeds (approx. 32 m/s) over long bowling spells (Burnett et al., 1995; Portus et al., 2000), which requires highly coordinated movement solutions (Worthington et al., 2013). These high speeds reduce the reaction time for batters, contributing to the success of fast bowlers (Bartlett et al., 1996). Over the past 25 years, more research has investigated the determinants of high release speed (Bartlett et al., 1996; Hanley et al., 2005; Middleton et al., 2016; Portus et al., 2004), with technique emerging as one of many contributing factors (Glazier et al., 2000). Fast bowling technique is complex, involving three main phases (Bartlett et al., 1996). The bowling action consists of the run‐up, bound and delivery stride (Bartlett et al., 1996). The delivery stride involves back foot contact (BFC), front foot contact (FFC) and ball release (BR). Faster bowlers convert linear momentum into angular momentum during the delivery stride, transferring it to the ball's linear velocity at release (King et al., 2016; Salter et al., 2007). Maintaining high release speeds requires bowlers to develop a versatile technique.

Fast bowling, with its complex nature and performance constraints, likely exhibits considerable variability amongst the various components of the action. Studies have identified BFC, FFC and BR as key stages in this action (Felton et al., 2019; Worthington et al., 2013). Variability in the timings between these stages of the bowling action is potentially indicative of movement variability along the kinetic chain. For example, Bootsma and van Wieringen (1990) used movement timing as a means of examining functional movement variability in table tennis drives. Therefore, examining variability of delivery stride movement timings can provide valuable insight into how fast bowlers adapt for consistent performance.

The dynamic nature of a fast‐bowling action provides an ideal task for extending research on functional movement variability. Existing literature primarily focuses on non‐run‐up tasks, such as basketball free throws (Button et al., 2003), baseball pitching (Matsuo et al., 2020) or golf shots (Morrison et al., 2014; Sayers et al., 2017). An exception is the work of Wilson et al. (2008) who examined the coordination variability of competitive triple jumpers. Skill improvement correlated with higher movement variability, which, in a similar vein to the previous research (Sayers et al., 2017), was suggested as having a functional role for coping with perturbations. However, the research had limitations such as a small sample size (Wilson et al., 2008) and lack of reported outcome variables. Further investigation is necessary to determine if trial‐to‐trial movement variability is associated with performance consistency.

While previous studies on fast bowling technique have explored variability over a bowling spell (Burnett et al., 1995; Schaefer et al., 2018), this research lacks the ball‐to‐ball detail to understand performance maintenance. Phillips et al. (2012) helped to address this limitation by examining changes in fast bowling accuracy over successive deliveries. Skilled cricket bowlers demonstrated superior accuracy maintenance despite varying delivery types. However, the research did not examine how skilled bowlers adapted movements for consistent outcomes despite being interpreted as functional adaptations (Phillips et al., 2012). The authors recommended exploring the relationship between movement variability and performance outcomes in future research. Furthermore, delivery speed has been recognized as a key contributor to the successful performance of fast bowlers in cricket (Worthington et al., 2013). Building on the previous work that focussed on delivery accuracy, the present study explores whether movement variability contributes to consistent delivery speed in cricket fast bowling.

The purpose of this study was to determine whether movement variability during a highly dynamic and fast‐paced skill contributes to a consistent performance outcome. To achieve this aim, this study examined how movement timings variability in the final delivery phases of the fast‐bowling action for skilled performers contributes to a consistent delivery speed. Building upon the concept of functional variability observed in other sports (Sayers et al., 2017), it was hypothesized that the bowlers would exhibit greater movement timing variability than delivery speeds, indicating that bowlers made adjustments to maintain a consistent bowling speed. Similar to findings in other activities (Bootsma & Peper, 1992; Bootsma & van Wieringen, 1990), it was also hypothesized that variability in the latter phase (FFC‐BR) would be less than in the earlier phase (BFC‐FFC), indicating bowlers' progressive reduction of movement variability toward ball release.

## 2 METHOD

### 2.1 Participants

The participants were 92 male fast bowlers (M age = 16.9 ± 1.3 years) who were members of their state youth pathway program. Bowlers in this program were selected based on their potential to progress to state representation at youth national championships. The research received institutional ethics approval (approval no. HEC20058).

### 2.2 Procedure

The procedure followed a standardized bowling skill test that has been embedded within the national talent identification structure (similar to that used by Phillips et al., 2012). All bowlers were instructed to perform their usual pre‐game stretching and warm‐up routine. After the warm‐up, bowlers bowl at match intensity for three overs (18 deliveries) to a batsman in an outdoor‐netted turf pitch practice facility. Due to time constraints and the large sample size (92 bowlers), analysis was limited to the footage of the first six of the 18 deliveries. Those first six deliveries were performed in the same order for each participant and consisted of two full‐length deliveries (pitching less than 6 m from the batter), two good length deliveries (pitching between 6 and 8 m from the batter) and two short length deliveries (pitching greater than 8 m from the batter; see also Phillips et al., 2012). While attempting to maintain the match intensity, bowlers knew they were being assessed on delivery speed and accuracy simultaneously with each delivery. Given that the research focussed on delivery speed, accuracy was not recorded, but coaches assessed the execution of each delivery to ensure adherence to the required delivery type. The different delivery types were used to encourage bowlers to execute deliveries typically required in competition for success, thereby helping to improve the representativeness of the experiment. Bowlers had no rest periods during the assessment but completed each session with another bowler, allowing for a slightly longer break between deliveries compared to completing the assessment by themselves. Delivery speed was measured at the instant of ball release using a radar gun with an accuracy of ±3% (Stalker Pro II, 34.7 GHZ). Given that previous studies reporting average delivery speeds of around 32 m/s (Burnett et al., 1995; Portus et al., 2000), the measurement error for delivery speed is approximately ± 0.96 m/s. With a similar model radar gun, for the same positioning (zero degree offset) as the current study, a strong correlation (*r* = 0.99, *p* < 0.001) was found between the radar gun and 3D motion capture (Smith & Burke, 2021). Consistent with previous studies, the radar gun was placed 30 m behind the bowling crease, directly in line with middle stump, and 1.5 m high (Sims et al., 2021). Motion data were collected with a standard video camera (Basler Aca2000 ‐ 165uc) positioned alongside the radar gun at a height of 1.7 m, operating at 100 frames per second. The camera was configured to automatically adapt to varying light conditions while ensuring the necessary shutter speed for clear image capture in each frame.

### 2.3 Data processing

The image processing and analyses were completed using Kinovea (Version 0.8.26). The instants of back foot contact (BFC), front foot contact (FFC) and ball release (BR) were identified manually. BFC was the first frame in which the back foot contacted the ground. FFC was the first frame in which the front foot contacted the ground. BR was the last frame in which the ball was in contact with the bowler's hand. These timepoints for each delivery were imported into Microsoft Excel (version 2210; Microsoft Corporation). The differences between the timepoints were used to calculate the movement timings between the first (BFC‐FFC) and second (FFC‐BR) phases of the bowling action. Each delivery involved calculating the difference between BFC and FFC for the first movement phase and between FFC and BR for the second movement phase. This process was completed 6 times for all 92 bowlers, resulting in 552 balls included in the analysis. The outcome variable was delivery speed, measured in m/s. For 11 deliveries, delivery speed did not register on the radar gun. The mean and standard deviation of the six deliveries for each bowler were calculated.

### 2.4 Data analysis

The intra‐individual variability of movement timings (BFC‐FFC and FFC‐BR) and delivery speed were determined using the coefficient of variation (CV). The intra‐individual CVs were determined for each individual participant by dividing their standard deviation by their mean for each of the dependent variables (i.e., BFC‐FFC, FFC‐BR and delivery speed), and then expressing these values as percentages. Using these values, separate group means were calculated for each of the three dependent variables (i.e., BFC‐FFC, FFC‐BR and delivery speed; see Table 1).

**TABLE 1:** Mean, standard deviation and coefficient of variation for delivery stride phases and delivery speed.

Variable	Mean ± SD	CV (%)
BFC‐BR (ms)	323 ± 90.2	2.5
BFC‐FFC (ms)	197 ± 58.8	4.7
FFC‐BR (ms)	126 ± 37.2	4.8
Delivery speed (m.s^−1^)	33 ± 2.0	2.0

Abbreviations: BFC, Back foot contact; BR, Ball release; FFC, Front foot contact.

Due to violations of the assumption of normality, a Friedman's 2‐way analysis of variance (ANOVA) was conducted in SPSS (IBM, Armonk, NK, USA) to examine whether different length deliveries (good, short and full) caused a change in the variability (CV) of delivery stride movement timings or delivery speed. Alpha was set at *p* < 0.05.

CV ratios were used to compare the variability among BFC‐FFC and FFC‐BR (FFC‐BR divided by BFC‐FFC), BFC‐FFC and delivery speed (BFC‐FFC divided by delivery speed) and FFC‐BR and delivery speed (FFC‐BR divided by delivery speed). The comparison of BFC‐FFC and FFC‐BR aimed to assess the direction of variability change in movement timings from the first phase (BFC‐FFC) to the second phase (FFC‐BR). This statistical analysis was conducted in Microsoft Excel (version 2210; Microsoft Corporation). Similar to the findings in other motor skill studies (Bootsma & Peper, 1992; Bootsma & van Wieringen, 1990; Morrison et al., 2014), a decrease in variability from the first phase to the second phase would indicate bowlers' efforts to reduce movement variability in the final phase of the action. The comparison between the two movement phase timings (BFC‐FFC and FFC‐BR) and delivery speed were used to determine whether the variability in the movement timings were more or less than the variability recorded for delivery speed. Less variability in the delivery speed compared to that observed for the movement timings would suggest that the bowlers were attempting to achieve a consistent delivery speed by varying their movements. For all comparisons, a CV ratio between 0.9 and 1.1 was considered trivial, a CV ratio >1.1 indicated substantially more variability, and a CV ratio <0.9 indicated substantially less variability (Crowther et al., 2018; Drinkwater et al., 2007).

## 3 RESULTS

**FIGURE 1:**
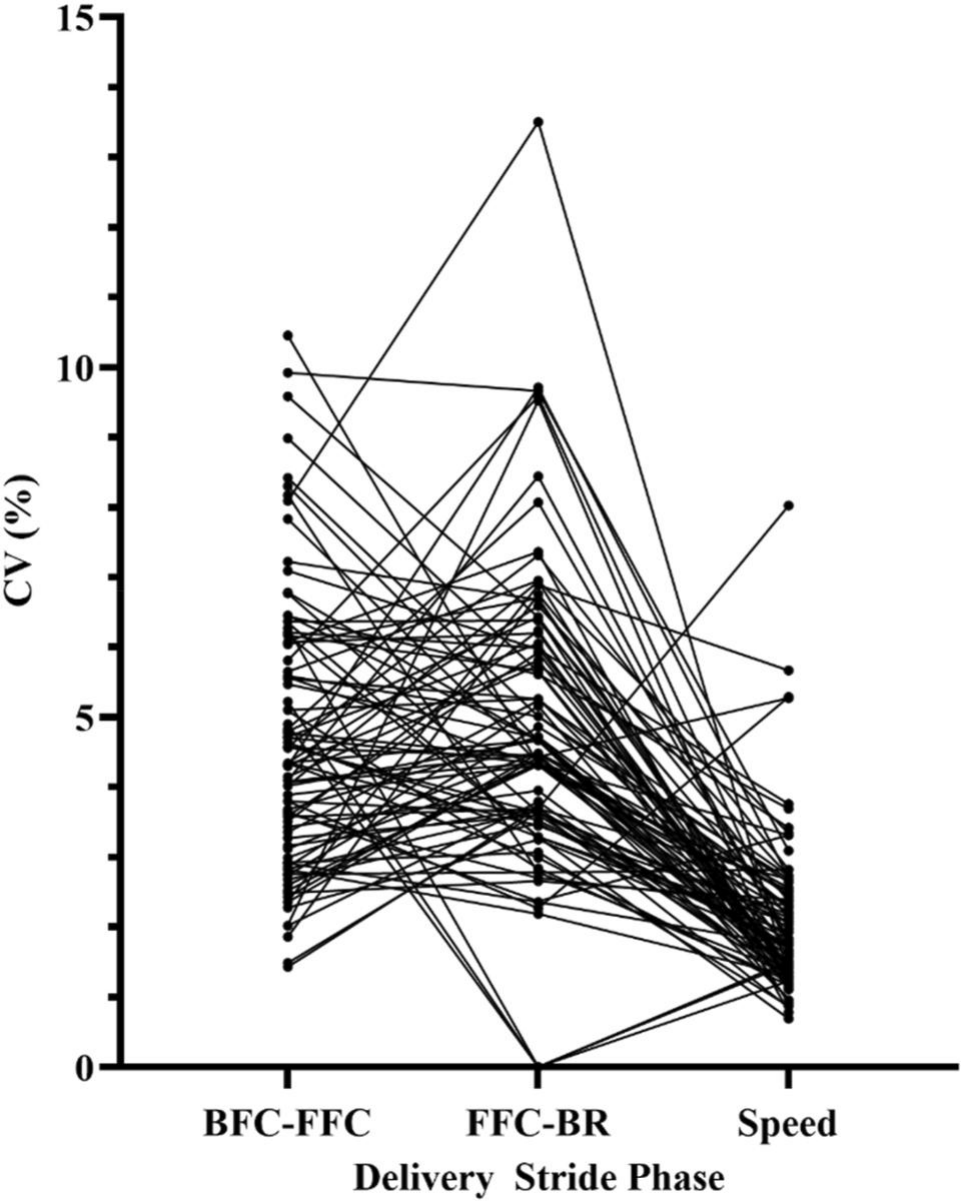
Intra‐individual coefficient of variation values for each bowler across delivery stride phases and delivery speed.

Greater variability in the delivery stride compared to release speed was indicated in the CV ratios comparing BFC‐FFC to delivery speed (CV ratio = 2.31), FFC‐BR to delivery speed (CV ratio = 2.38) and BFC‐BR to delivery speed (CV ratio = 1.25; Table 1). This showed that the bowlers exhibited substantially more variability between each delivery stride movement timing compared to the amount of variability in the performance outcome measure of delivery speed (Figure 1). The CV ratio comparing BFC‐FFC to FFC‐BR was 0.97, which was considered trivial. The latter result indicated the variability in the movement timings did not change between the first (BFC‐FFC) and second (FFC‐BR) phases of the movement.

Friedman's ANOVA revealed no significant differences in CV across the different bowling lengths (good, short and full) for BFC‐FFC, *χ* ^2^ (2) = 1.34, *p* = 0.51, FFC‐BR, *χ* ^2^ (2) = 4.46, *p* = 0.11, BFC‐BR, *χ* ^2^ (2) = 0.57, *p* = 0.75 or delivery speed, χ^2^ (2) = 0.58, *p* = 0.75. These results indicated that the intended bowling length did not cause a significant change in delivery stride parameters or delivery speed.

## 4 DISCUSSION

This study aimed to extend previous work on functional variability (Phillips et al., 2012; Sayers et al., 2017; Wilson et al., 2008) by examining the contribution of delivery stride movement timings variability to consistent bowling speeds. The results supported the first hypothesis, showing greater variability in the bowlers' delivery stride movement timings (BFC‐FFC and FFC‐BR) compared to delivery speed variability. While traditional motor control theories interpret movement variability as noise and system error (Adams, 1971; Schmidt, 1975), an alternative interpretation suggests that the variability exhibited by the bowlers in their movement timings may have been functional in nature (see also Davids et al., 2003; Morrison et al., 2014; Wilson et al., 2008). In other words, if the variability in movement timings of the delivery stride reflected the bowlers attempting to adapt their movements to produce consistent delivery speeds, it could be considered functional (Bartlett et al., 2007; Davids et al., 2003). Indeed, as bowlers become more experienced, they improve their capability to functionally vary their movements to maintain a consistent outcome (Phillips et al., 2012).

Previous research has shown that variability can be beneficial for performers in adapting during the movement and between trials (Bootsma & van Wieringen, 1990; Fleisig et al., 2009). The results reported here are the first to provide preliminary evidence suggesting that cricket fast bowlers may use functional movement variability to maintain their delivery speed. However, these results are based upon inferences derived from movement phase timing variability, which may be influenced by factors such as variations in foot placement, running speed, movements of the opposing batter or the bowler's physical and emotional state (Davids et al., 2003; Langdown et al., 2012). The influence of constraints on movement variability is an important topic for future research, particularly in the context of cricket fast bowling (Phillips et al., 2012). Future research should aim to confirm and expand upon these findings by investigating the influence of task‐specific constraints on movement variability and performance outcomes in cricket fast bowlers.

The capability of skilled cricket fast bowlers to adapt to changing task constraints to maintain a consistent performance outcome has been reported previously by Phillips et al. (2012). However, in contrast to the present study, these researchers also found that this trend was mainly associated with the more highly skilled bowlers, whereas for bowlers who were a similar standard to those in the present research (junior pace squad members), these functional adaptations were not evident. Perhaps, in line with the present study, junior bowlers have sufficient skill to maintain consistent delivery speeds, while their capacity for consistent accuracy, as shown by Phillips et al. (2012), requires further development. Alternatively, it is possible that differences in methodological designs between the present research and that of Phillips et al. (2012) contributed to the differences in the results. Phillips et al. (2012) used an indoor synthetic pitch with a projected image of a batter, whereas the present research used an outdoor cricket pitch with a real batter. The other obvious difference is the fact the present study focussed on assessing the relationship between movement timing variability and delivery speed, whereas Phillips et al. (2012) did not directly measure bowlers' movements and instead examined accuracy variability under varying task constraints. Hence, future studies should measure how movement variability contributes to performance of both accuracy and delivery speed across different playing levels. Nevertheless, the present results add to the extant literature by suggesting that functional movement variability may contribute to achieving a consistent performance outcome in multiarticular tasks performed while running at high speed (see also Wilson et al., 2008).

The predicted reduction in movement timing variability from the first movement phase (BFC‐FFC) to the second (FFC‐BR) revealed only trivial differences. In previous research, expert performers have been shown to progressively reduce the amount of movement variability as the performer approaches the endpoint of the action (Bootsma & van Wieringen, 1990; Horan et al., 2011; Matsuo et al., 2001). The greater variability in the earlier phases of the movement is believed to enable the final moment of the action to be more precise so the desired outcome can be achieved with greater success (Bootsma & Peper, 1992). However, as shown by Button et al. (2003) in basketball free throw shooting, movement variability does not always reduce toward the endpoint of the action but can in fact increase in a proximal‐to‐distal direction. The results of Button et al. (2003) revealed that movement variability observed at the elbow joint for the better free throw shooter at the moment of ball release (*SD* = 1.8°) was considerably less than the variability at the wrist joint (*SD* = 5.1°), which was the endpoint of the action. These differences suggest that the manner in which movement variability manifests is likely to be dependent upon the nature of the task itself. In the present study, the measurement methods that were used to assess movement timings may explain why a reduction in timing variability toward the end point of the delivery was not evident. Previous research has examined variability by measuring the movement of specific body parts or specific pieces of equipment (Bootsma & van Wieringen, 1990), whereas the measurements used in the present research used movement timings that encapsulated the movement of several different body parts. To further examine movement variability in different motor tasks, comprehensive technical analysis is required, including three‐dimensional motion tracking at high sampling frequencies for precise measurement of joint variability in kinematic and kinetic factors (Phillips et al., 2012). For applied studies looking to assess movement timings, the number of degrees of freedom the player is required to resolve is also an important consideration for studying movement timing variability (Button et al., 2003).

This study has practical implications for coaches and sports scientists working with fast bowlers in cricket. Traditional perspectives of skill acquisition would advocate for practice repetitions to produce a consistent bowling action (Bartlett et al., 2007; Davids et al., 2003; Langdown et al., 2012). However, the present study provide data to suggest that bowlers may use functional movement variability in their bowling action to adapt their movement patterns to achieve consistent performance outcomes (see also Phillips et al., 2012). From an applied perspective, presenting bowlers with a variety of performance scenarios can facilitate adaptability (Phillips et al., 2012). Indeed, constraints‐led approaches to training manipulate individual, task and environmental constraints to encourage athletes to seek adaptive movement solutions, encouraging them to explore different ways of achieving the same task goal (Chow et al., 2022; Phillips et al., 2012; Renshaw et al., 2010, 2019). Coaches could design more variable training sessions so that bowlers discover new and effective ways of adapting to various constraints (Chow et al., 2022; Phillips et al., 2012).

This study presents preliminary evidence of functional movement variability in fast bowlers. While the applied nature of this study limited the comprehensiveness of data collection, it was possible to obtain a large sample size of 92 bowlers. Future studies could explore the relationship between functional movement variability and fast bowling performance by investigating both release speed and accuracy as performance outcomes. Moreover, investigating the effect of different constraints on movement pattern variability can help inform more effective ways of designing training sessions to facilitate adaptive movement outcomes for fast bowlers (Phillips et al., 2012).

## 5 CONCLUSION

The results from this study show how fast bowlers in cricket may use functional movement variability to achieve a consistent delivery speed. These findings challenge conventional notions of skill acquisition, which commonly advocated minimizing movement variability as a prerequisite for successful performance outcomes (Adams, 1971; Schmidt, 1975). This study suggests that while fast bowlers exhibited variability in their movement timings, this variability likely played a functional role in assisting them adapting to task constraints and achieving consistent performance outcomes. For coaches and sports scientists, designing training sessions that involve variable practice could encourage bowlers to discover new and effective ways of adapting to various constraints.

## CONFLICT OF INTEREST STATEMENT

The authors have no conflicts of interest to disclose.
